# LP533401 treatment alters vitamin K metabolism, bone Gla-OC/Glu-OC ratios, and improves mineral status of the skeleton in rats with chronic kidney disease

**DOI:** 10.3389/fphar.2026.1793663

**Published:** 2026-09-04

**Authors:** Magdalena Kopańko, Beata Sieklucka, Dariusz Pawlak, Magdalena Zabłudowska, Katarzyna Sokołowska, Urszula Łebkowska, Krystyna Pawlak

**Affiliations:** 1 Department of Monitored Pharmacotherapy, Medical University of Bialystok, Bialystok, Poland; 2 Department of Pharmacodynamics, Medical University of Bialystok, Bialystok, Poland; 3 Department of Radiology, Medical University of Bialystok, Bialystok, Poland

**Keywords:** bone mineralization, chronic kidney disease, LP533401, serotonin, vitamin K

## Abstract

**Background:**

Chronic kidney disease-mineral and bone disorder (CKD-MBD) is associated with impaired bone mineralization and vitamin K (VK) deficiency. Peripheral serotonin (5-HT) adversely affects bone integrity, but its relationship with VK metabolism remains unknown. In this study, we investigated the effects of LP533401 (LP), a small molecule inhibitor of peripheral 5-HT synthesis in the gut, on VK metabolism, VK-dependent proteins (VKDPs), and mineral status of the skeleton in CKD rats.

**Methods:**

Serum levels of phylloquinone (VK1), menaquinone 4 and 7 (MK4, MK7), serum and bone VKDPs concentrations, bone VK cycle genes expression, and mineral status of the skeleton were determined in CKD rats treated with vehicle or LP at the dose of 30 or 100 mg/kg daily.

**Results:**

LP treatment altered VK metabolism by restoring circulating VK1 levels and promoting MK7 over MK4 synthesis. At the molecular level, LP treatment modulated the expression of VK cycle genes (*UBIAD1, VKORC1, GGCX*) and improved bone γ-carboxylation efficiency, particularly in trabecular bone. LP at 30 mg/kg most effectively enhanced bone mineral parameters of the skeleton, while the 100 mg/kg dose had site-specific effects, restoring mainly cortical bone mineralization. Correlations between MK7 and bone densitometric parameters highlight its functional role in restoring mineral homeostasis.

**Conclusion:**

LP treatment modulates VK metabolism, VKDPs levels in bone and serum, and improves bone mineralization in the rat CKD model. This study provides a new perspective on the therapeutic potential targeting the modulation of peripheral serotonin and VK-dependent signaling to improve bone mineralization, which is impaired in CKD-MBD.

## 1 Introduction

Chronic Kidney Disease (CKD), referred to as persistent structural or functional impairments of the kidneys (Kidney Disease, 2024; Kidney Disease, 2017), impacts an expanding share of the population, thereby posing a significant and escalating challenge for the healthcare system (Kovesdy, 2022; Qin et al., 2024). Currently, it is estimated that roughly 850 million people suffer from CKD (Jager et al., 2019).

Among the complications of CKD associated with increased mortality are mineral and bone disorders (CKD-MBD) (Kidney Disease, 2017), which disrupt bone metabolism, resulting in impaired mineralization and an elevated fracture incidence (Pimentel et al., 2017). Compared with the general population, low bone mineral status was more frequent in CKD (Leonard et al., 2019). Throughout CKD, complex alterations occur in various factors involved in the regulation of bone mineralization, including calciotropic hormones, the deficiency of minerals (Hu et al., 2022; Ketteler et al., 2025), and micronutrients, especially vitamin K (VK) (de Oliveira et al., 2020; Bellone et al., 2022).

Vitamin K comprises a group of lipid-soluble vitamins that are classified into two primary forms: vitamin K1 (VK1), or phylloquinone, and vitamin K2 (VK2), a collective designation for menaquinones (MKs). Among VK2, the MK4 and MK7 forms are involved in bone remodeling and bone health (Chen et al., 2022; Popa et al., 2021). VK1 is primarily obtained from leafy green vegetables and plant oils, whereas VK2 originates from animal products and is synthesized by fermentative bacteria (Popa et al., 2021; Dupuy et al., 2025). Moreover, MK4 may be produced through the enzymatic conversion of phylloquinone by UbiA prenyltransferase domain-containing protein-1 (UBIAD1) (Chen et al., 2022; Shea and Booth, 2022). The biological activity of VK is maintained by the vitamin K cycle – a metabolic pathway responsible for its regeneration, which enables its continuous participation in essential physiological processes (Bus and Szterk, 2021). The cycle begins with the reduction of VK quinone to its active form, hydroquinone, catalyzed by quinone reductase. In this form, VK functions as a cofactor for the enzyme γ-glutamyl carboxylase (GGCX) in the γ-carboxylation of proteins. Following this process, VK is converted into its epoxide form, which is subsequently regenerated back to the quinone form by the enzyme vitamin K epoxide reductase complex subunit 1 (VKORC1) (Bryshten et al., 2024). The key VK-dependent proteins (VKDPs) directly involved in bone mineralization are osteocalcin (OC) and matrix Gla protein (MGP). Upon γ-carboxylation, these proteins acquire Gla domains, which are essential for their biological activity and enable them to bind hydroxyapatite, the main inorganic component of bone (Komori, 2020; Zhang et al., 2019). Vitamin K deficiency is frequently observed in patients with CKD (Kremer et al., 2021; Silaghi et al., 2019; Fusaro et al., 2017; Neradova et al., 2022). VK2 supplementation is generally believed to improve bone mineral density (BMD) in older adults, especially postmenopausal women (Aaseth et al., 2024). However, data on the relationship between VK levels and bone health in CKD patients are very limited (Kohlmeier et al., 1997; Ghelichi-Ghojogh et al., 2025). To date, only one clinical trial has examined the effect of MK-7 supplementation on the BMD of the radius and lumbar spine in hemodialyzed patients. The accelerated BMD loss of the 1/3 distal radius was observed compared with placebo, whereas the decrease in lumbar spine BMD was prevented with MK-7 supplementation (Levy-Schousboe et al., 2023).

Our previous experiments, performed on young rats with CKD, have indicated the adverse effects of elevated peripheral serotonin (5-HT) levels on bone metabolism and strength (Pawlak et al., 2016; Pawlak et al., 2017). We also demonstrated that inhibition of 5-HT synthesis in the gut through a tryptophan hydroxylase 1 inhibitor (LP533401) had therapeutic potential to restore impaired mineral status and strength of the femur in an experimental model of CKD (Pawlak et al., 2018). Recently, we demonstrated for the first time that the bone tissue of rats expresses the enzymes enabling the conversion of VK1 into menaquinone forms, as well as VK recycling (Ziemińska et al., 2022). The present study is the continuation and extension of our previous findings. As both peripheral 5-HT and VK metabolites can influence bone mineralization in our CKD model (Pawlak et al., 2016; Pawlak et al., 2017; Pawlak et al., 2018; Ziemińska et al., 2022), the current study focuses on determining the effect of LP533401 therapy on endogenous VK metabolism and VK-dependent bone mineralization in rats with experimental CKD.

## 2 Materials and methods

### 2.1 Animal model and experimental design

During the entire experiment, 74 male Wistar rats stayed in the Experimental Medicine Center of the Medical University of Bialystok (Poland). Animals were maintained under standard conditions (conventional laboratory cages, temperature 24 °C, 12-h light/dark cycle, and 50% humidity) with continuous access to sterilized tap water and standard rodent chow (Ssniff R/M-H; Ssniff Spezialdiäten GmbH, Soest, Germany) composed of 1% calcium, 0.7% phosphorus, 19% protein, 1,000 IU of vitamin D3/kg, and 5 mg of vitamin K/kg. Experimental CKD was induced by the surgical procedure of subtotal nephrectomy according to Švíglerová et al., 2010. Briefly, 4-week-old rats (n = 64) underwent a two-step surgical resection of the 5/6 kidney according to a standard protocol (Pawlak et al., 2016). The control group (CON, n = 10) underwent a sham operation consisting of laparotomy with bilateral renal exposure and gentle manipulation of the kidneys, including decapsulation, and closure of the abdominal cavity without removal of renal tissue (Pawlak et al., 2016). All postoperative procedures, including analgesia, monitoring, and housing conditions, were identical in sham-operated and nephrectomized animals to control for non-specific effects of surgery. After 12 weeks of CKD development, nephrectomized rats were randomly assigned to four experimental groups (n = 16 in each): untreated (CKD), vehicle-treated (VEH; polyethylene glycol and 5% dextrose in a 40:60 ratio), and two groups treated with LP533401 [(2S)-2-amino-3-(4-(2-amino-6-(2,2,2-trifluoro-1-(3′-fluorobiphenyl-4-yl)ethoxy)pyrimidin-4-yl)phenyl)propanoic acid; Dalton Pharma Services, Canada] at doses of 30 mg/kg [LP (30 mg)] and 100 mg/kg [LP (100 mg)], respectively. Both LP and VEH were administered once daily by oral gavage for 8 weeks. The untreated CKD and control groups received sterile water by oral gavage using the same schedule, as described previously (Pawlak et al., 2018). Randomization was performed using key variables, including serum creatinine concentration and body weight, to ensure balanced allocation. The randomization sequence was generated by an independent investigator (an employee of the Experimental Medicine Center) not involved in surgical procedures, treatment administration, or outcome assessment. Animals were assigned unique identification numbers, and outcome assessors were blinded to group allocation whenever feasible. During the experiment, two animals died (one in the VEH group and one in LP (100 mg) group, and one rat from the VEH group was excluded from subsequent analyses due to severe renal failure (as previously described in detail (Pawlak et al., 2018). Therefore, the final numbers of animals included in the analysis were: CON (n = 10); CKD (n = 16); VEH (n = 14); LP (30 mg) (n = 16); and LP (100 mg) (n = 15); (Pawlak et al., 2018). Schematical presentation of changes in basic parameters across all experimental groups has been shown in Supplementary Table 1. The study protocol was approved by the Local Bioethics Committee in Bialystok (Resolution No. 29/2013, dated 24 April 2013).

### 2.2 Specimen collection and storage

In the present study, we used biological material collected during our previous experiment (Pawlak et al., 2018). Following the induction of anesthesia, blood was collected by cardiac puncture. Serum was obtained by centrifugation at 400 × g for 10 min and subsequently stored at −80 °C. The tibiae and lumbar spine (L1–L5) were cleared of surrounding tissues, wrapped in gauze moistened with physiological saline, and stored at −20 °C for densitometric analysis. Samples were thawed to room temperature before densitometric measurements. The femurs were cleared and immediately frozen at −80 °C to prepare tissue homogenate and perform genetic testing.

### 2.3 Bone mineral status analysis using dual-energy x-ray absorptiometry (DXA)

Upon equilibration to room temperature, the right tibiae and lumbar spine (L1–L5) were scanned using a Horizon QDR Series X-ray Bone Densitometer (Hologic Inc., Bedford, MA, United States) with integrated small animal analysis software (Pawlak et al., 2018). All measurements were performed by a single qualified radiologist and included bone mineral density (BMD, mg/cm^2^), bone mineral content (BMC, mg), and bone mineral area (BMA, cm^2^). The machine was calibrated daily with a phantom provided by the manufacturer.

### 2.4 Liquid chromatography-tandem mass spectrometry (LC-MS/MS)

Serum concentrations of Vitamin K forms (VK1, MK4, and MK7) were measured by the Laboratory of Perlan Technologies Polska (Gdynia, Poland) using LC-MS/MS, as previously described in detail (Ziemińska et al., 2022). The assay was performed using an Agilent Technologies 1260 LC system coupled with a G6470A Triple Quadrupole LC-MS (Agilent Technologies, Santa Clara, CA, United States) and operated with Agilent MassHunter Workstation Software (version B.10.00).

### 2.5 Preparation of bone tissue homogenates

To determine the VKDPs in bone tissue, homogenates of trabecular and cortical bone, isolated from the femoral distal end and femoral diaphysis, respectively, were prepared. The segments of bone tissue were weighed, and after washing out with physiological saline to eliminate the remaining available bone marrow, were crumbled and homogenized in the cold 50 mM potassium phosphate buffer (pH = 7.4) using a high-performance homogenizer (Ultra-Turrax T25; IKA, Staufen, Germany) equipped with a stainless-steel dispersing element (S25N-8G; IKA). The buffer volume was converted to the mass of each sample to obtain a 10% tissue homogenate. The supernatant was obtained by centrifugation of 10% homogenates for 10 min at 700 × g at 4 °C and stored at −80 °C until analysis.

### 2.6 Serum and bone levels of vitamin K-dependent proteins

VKDPs were determined using enzyme-linked immunosorbent assays (ELISA), following the manufacturer’s instructions (Ziemińska et al., 2022). The ELISA Kit from MyBioSource Inc. (San Diego, CA, United States) was used to quantify undercarboxylated Gla matrix protein (ucMGP). Undercarboxylated osteocalcin (Glu-OC) was measured using the Rat Glu-Osteocalcin High Sensitive EIA, carboxylated osteocalcin (Gla-OC) using the Rat Gla-Osteocalcin High Sensitive EIA (Takara Bio Inc., Shiga, Japan), and total osteocalcin using the Rat Osteocalcin ELISA kit (Immunotopics Inc., San Clemente, CA, United States). The obtained Gla-OC and Glu-OC results were corrected for the protein concentration in the trabecular and cortical regions, and the Gla-OC/Glu-OC ratio was calculated.

### 2.7 Quantitative real-time polymerase chain reaction (qRT-PCR)

The GeneJET RNA Purification Kit (Thermo Scientific, Lithuania) was used to extract total RNA from bone tissue. Subsequently, RNA concentration and purity were measured using a Thermo Scientific NanoDrop 2000 spectrophotometer, and RNA integrity was assessed with an Agilent 2100 Bioanalyzer (Agilent Technologies, United States). Only samples with acceptable RNA integrity were used for further analysis. Reverse transcription was performed using 1 μg of total RNA and the RevertAid™ First Strand cDNA Synthesis Kit (Fermentas, Canada), following the manufacturer’s protocol. Quantitative real-time PCR (qRT-PCR) was carried out using the Stratagene Mx3005P QPCR System (Agilent Technologies, United States) with SG qPCR Master Mix (2x; EURx, Poland). Amplification reactions were performed under standard cycling conditions recommended by the manufacturer. Relative gene expression levels were quantified using the comparative Ct (ΔΔCt method). Glyceraldehyde 3-phosphate dehydrogenase (GAPDH) was employed as a housekeeping gene to normalize expression levels. A pooled cDNA sample from the control group was used as the calibrator in ΔΔCt calculations. Primers were designed using Primer-BLAST software (Primer3 program). The primer sequences were (5′-3′ forward-reverse): *VKORC1* (5′-TCC​CGC​GTC​TTC​TCC​TCT​C-3′; 5′-CCA​ACG​TCC​CCT​CAA​GCA​AC-3′; amplicon size: 150 bp), *GGCX* (5′-GGA​TGC​TGA​CTG​GGT​TGA​GG-3′; 5′-GCT​CCT​CCG​ACA​ACA​CTA​GC-3′; amplicon size: 89 bp) and *UBIAD1* (5′-GCT​GTG​TGT​GTG​CTG​CTT​AC-3′; 5′- CCC​AGT​GCC​ACG​TAC​TTG​AA-3′; amplicon size: 133 bp). Primer specificity was verified by melting curve analysis following amplification, confirming the presence of a single specific product for each assay. Amplification efficiency for each primer pair was determined using standard curves generated from serial dilutions of cDNA, and only assays with efficiencies between 90% and 110% were included in the analysis. All reactions were performed in triplicate (technical replicates).

### 2.8 Statistical analysis

Data normality was evaluated using the Shapiro-Wilk test. Variables with a normal distribution are presented as mean ± SD, while non-normally distributed variables are presented as median (interquartile range). One-way ANOVA was conducted for multiple group comparisons, with Duncan’s *post hoc* test applied to assess significant differences between groups (p < 0.05). Correlations between study variables were analyzed using Spearman’s rank correlation, and two-tailed p-values <0.05 were considered statistically significant. Statistica version 13 (StatSoft, Tulsa, OK, United States) was utilized for computations. Results were graphically presented using GraphPad Prism version 10.2.2 (GraphPad Software, United States) and Microsoft PowerPoint.

## 3 Results

### 3.1 Vitamin K metabolism in rats with CKD treated with LP533401

**FIGURE 1:**
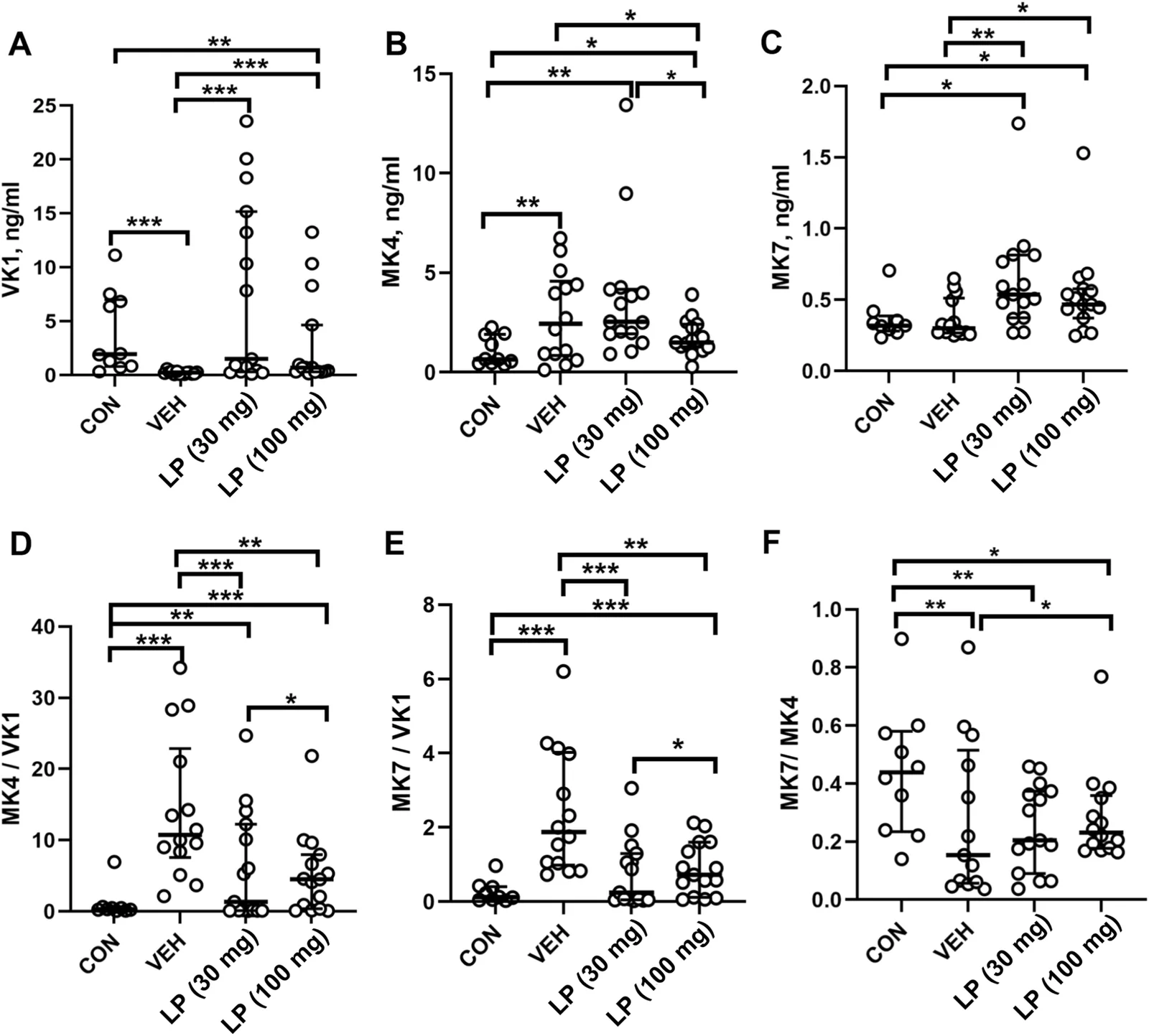
Serum Vitamin K1 **(A)**, MK4 **(B)**, MK7 **(C)** concentrations, and the ratios of MK4/VK1 **(D)**, MK7/VK1 **(E)**, and MK7/MK4 **(F)** in rats with chronic kidney disease (CKD) administered with vehicle (VEH) and treated with LP533401 (LP). Data are shown as median (interquartile range). *p < 0.05, **p < 0.01, ***p < 0.001; CON, controls; LP (30 mg), the group treated with LP at the dose of 30 mg/kg; LP (100 mg), the group treated with LP at the dose of 100 mg/kg; VK1, phylloquinone; MK4, Menaquinone 4; MK7, Menaquinone 7.

The transformation of serum phylloquinone (VK1) into menaquinone 4 (MK4) and MK7 forms, the expression of VK cycle genes in bone and the changes in VKDPs concentrations in bone and serum of rats with “clean” CKD were presented in our previous study (for details please see Ziemińska et al., 2022). As shown in Figure 1, serum VK1 levels were lowest in the VEH group compared to both CON and LP-treated animals. VK1 concentration decreased progressively with the use of increasing LP doses (Figure 1A). Conversely, MK4 levels were elevated in all experimental groups relative to CON, with the highest concentrations observed in the VEH and LP (30 mg) groups (Figure 1B). MK7 levels were also significantly increased in both LP-treated groups compared to CON and VEH (Figure 1C). The MK4/VK1 and MK7/VK1 ratios, the indices of VK1 conversion efficiency into their respective menaquinone forms, were highest in the VEH group, supporting our previous observations in “clean” CKD model (Ziemińska et al., 2022). These ratios were significantly higher in LP (100 mg) than in LP (30 mg) animals (Figures 1D,E). Consequently, the MK7/MK4 ratio was significantly lower in all CKD groups than in controls (Figure 1F). These data suggest that MK7 is the primary VK1-derived metabolite in LP-treated rats, whereas MK4 predominates in VEH-treated animals.

### 3.2 Expression of vitamin K cycle genes in the bone of rats treated with LP533401

**FIGURE 2:**
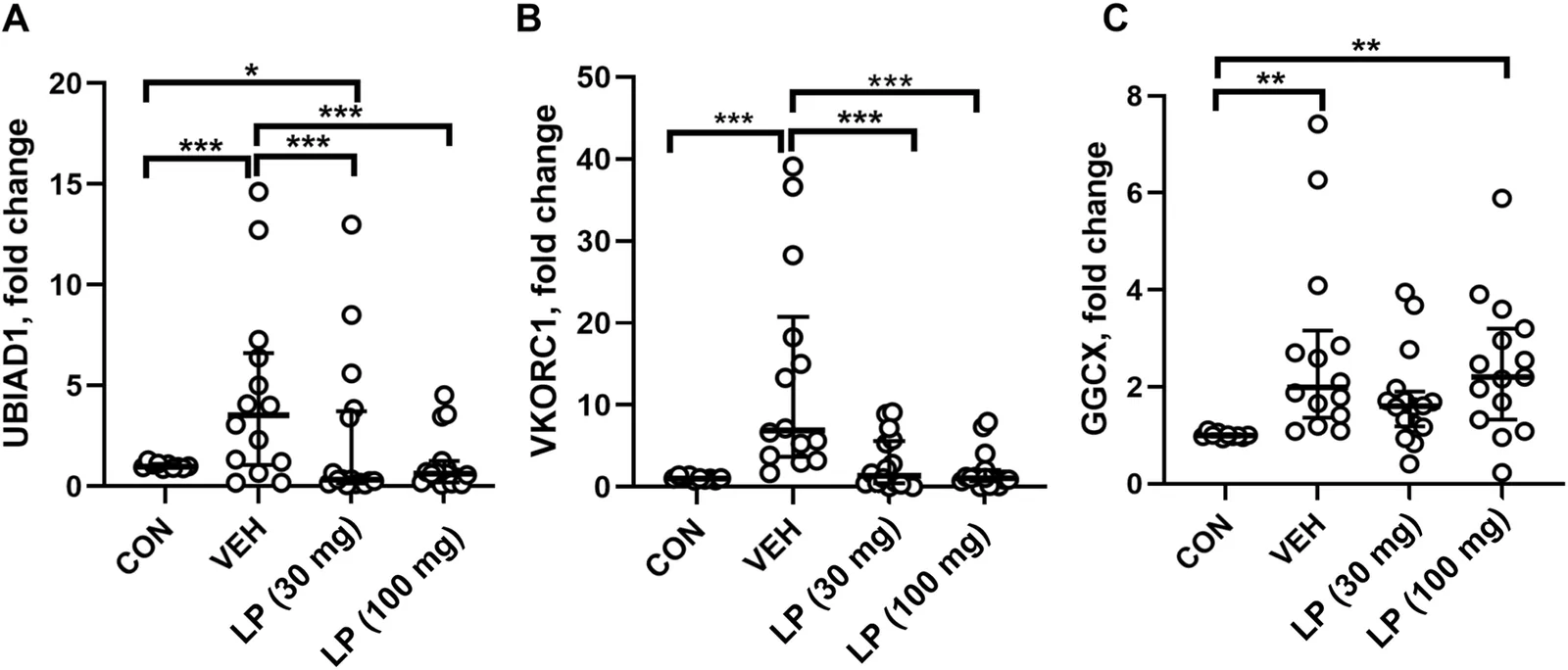
The expression of genes encoding vitamin K cycle enzymes: *UBIAD1* **(A)**, *VKORC1* **(B)**, and *GGCX* **(C)** in the bone of rats with chronic kidney disease (CKD) administered with vehicle (VEH) and treated with LP533401 (LP). Data are shown as median (interquartile range). *p < 0.05, **p < 0.01, ***p < 0.001; CON, controls; LP (30 mg), the group treated with LP at the dose of 30 mg/kg; LP (100 mg), the group treated with LP at the dose of 100 mg/kg; *UBIAD1*, UbiA prenyltransferase domain-containing protein 1; *VKORC1*, vitamin K epoxide reductase complex subunit 1; *GGCX*, the gamma-glutamyl carboxylase.

The animals administered with VEH showed the highest expression of *VKORC1* and *UBIAD1* compared to CON and LP-treated groups. *GGCX* mRNA levels were also increased in VEH in comparison with controls. *VKORC1* and *GGCX* gene expression was also increased in CKD group compared to healthy animals (Ziemińska et al., 2022). LP therapy reduced *VKORC1* and *UBIAD1* mRNA levels (Figures 2A,B) and normalized *GGCX* expression in the LP (30 mg) group. Interestingly, *GGCX* expression increased again at the higher LP dose (Figure 2C).

### 3.3 Effect of LP533401 treatment on vitamin K-dependent proteins in bone and serum

**FIGURE 3:**
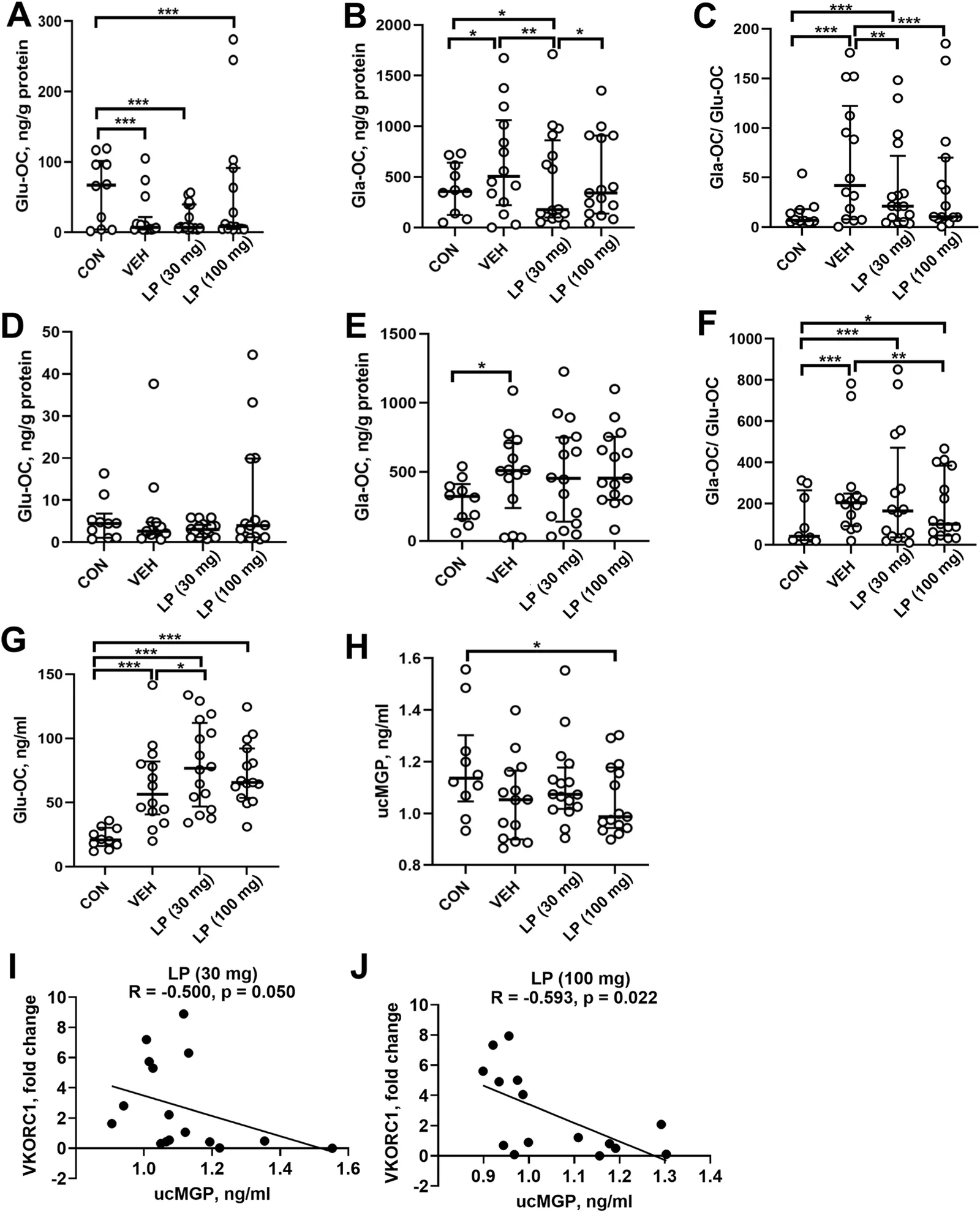
The levels of vitamin K-dependent proteins in trabecular **(A–C)**, cortical **(D–F)** bone compartments, and serum **(G,H)** of rats with chronic kidney disease (CKD) administered with vehicle (VEH) and treated with LP533401 (LP). The relationship between serum ucMGP concentration and bone *VKORC1* expression in LP (30 mg) **(I)**, and LP (100 mg) -treated animals **(J)**. Data are shown as median (interquartile range) - panels **(A–H)**, and Spearman’s correlation–panel **(I)**. *p < 0.05, **p < 0.01, ***p < 0.001; CON, controls; LP (30 mg), the group treated with LP at the dose of 30 mg/kg; LP (100 mg), the group treated with LP at the dose of 100 mg/kg; Glu-OC, undercarboxylated osteocalcin; Gla-OC, carboxylated osteocalcin; ucMGP, undercarboxylated Matrix Gla Protein; *VKORC1*, vitamin K epoxide reductase complex subunit 1.

Vitamin K functions as a GGCX cofactor in the carboxylation of osteocalcin (OC), converting Glu-OC to Gla-OC, which binds hydroxyapatite and contributes to bone mineralization (Atkins et al., 2009; Roy et al., 2001). In the current study, Glu-OC levels were significantly reduced in the homogenates of trabecular bone of all CKD groups compared to CON (Figure 3A). In cortical bone, these levels were slightly, though not significantly, lower (Figure 3D). Gla-OC levels and Gla-OC/Glu-OC ratios were significantly elevated in the VEH group relative to CON in both bone compartments (Figures 3B,C,E,F). Similar results were previously observed in CKD group (Ziemińska et al., 2022). LP (30 mg) treatment reduced Gla-OC in trabecular bone, though the Gla-OC/Glu-OC ratio remained higher than in controls. LP (100 mg) restored both parameters to levels comparable with CON (Figures 3B,C). In cortical bone, LP-treated groups showed moderately elevated Gla-OC levels relative to CON, with Gla-OC/Glu-OC ratios decreasing dose-dependently, yet remaining above control values (Figures 3E,F). Positive correlations were found between trabecular Glu-OC and Gla-OC in both VEH and LP groups (VEH: R = 0.566, p = 0.035; LP: R = 0.394, p = 0.028), but these correlations were absent in cortical bone.

Osteocalcin, its undercarboxylated form (Glu-OC), and uncarboxylated matrix Gla protein (ucMGP) reflect VK status and are used as markers of VK deficiency (Kremer et al., 2021; Silaghi et al., 2019; Fusaro et al., 2017; Caluwé et al., 2020). Previously, we showed that serum total OC levels were higher in all uremic groups, regardless of the use of LP or VEH (Pawlak et al., 2018). Serum Glu-OC levels were elevated in all CKD groups (Figure 3G; Ziemińska et al., 2022), whereas ucMGP levels remained unchanged in CKD (Ziemińska et al., 2022), VEH and LP (30 mg) groups, and were significantly reduced in LP (100 mg) compared to CON (Figure 3H). Notably, serum ucMGP levels in the LP (30 mg) and LP (100 mg) treated group were inversely correlated with *VKORC1* expression (Figures 3I,J; respectively), suggesting an important function of this gene in the gamma-carboxylation reaction (Tie and Stafford, 2016).

### 3.4 The effect of LP533401 on the mineral status of the skeleton in rats with CKD – the role of VK metabolites in bone mineralization

**FIGURE 4:**
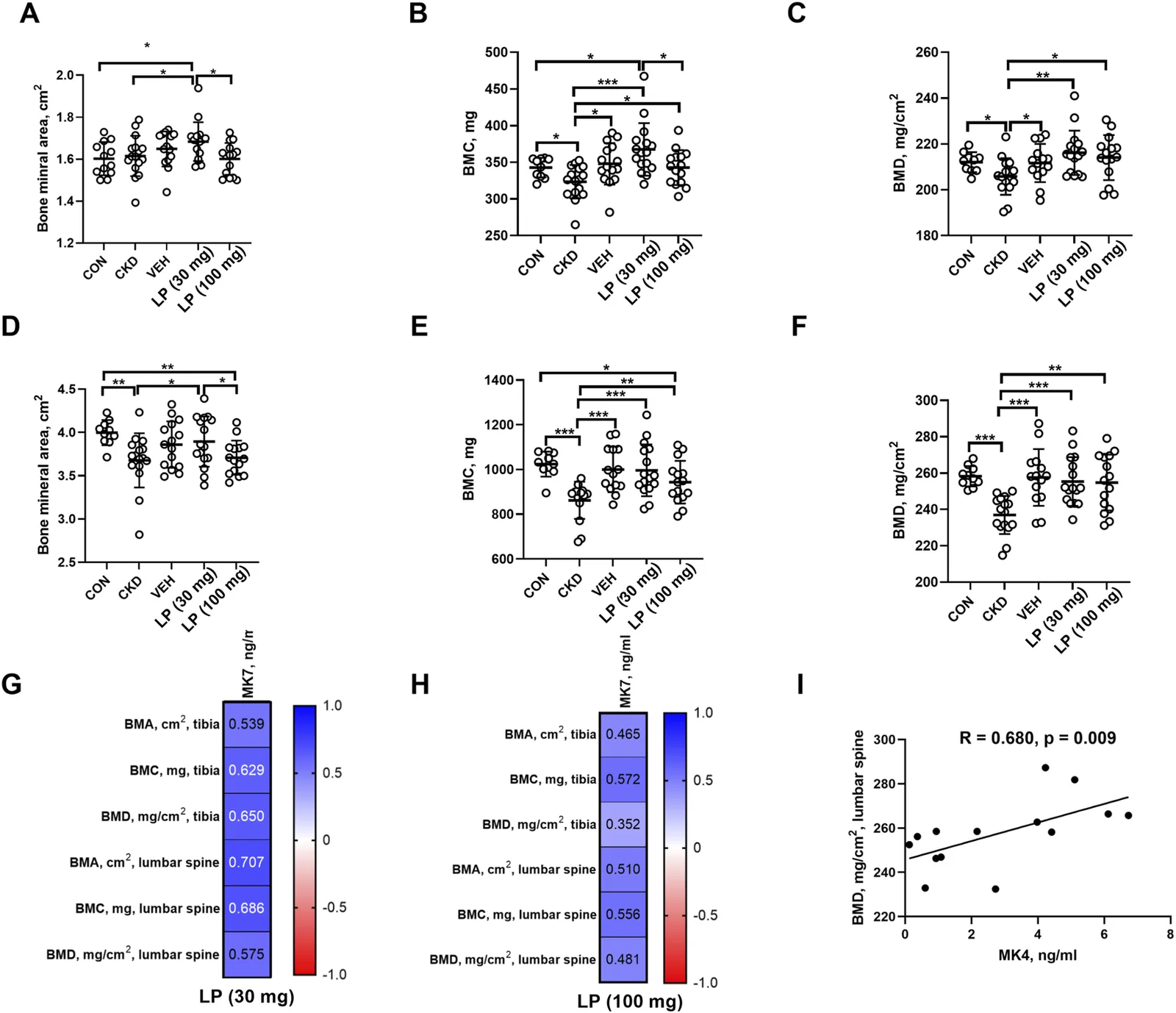
The mineral status of the tibia **(A–C)** and lumbar spine **(D–F)** of rats with chronic kidney disease (CKD) administered with vehicle (VEH) and treated with LP533401 (LP). The relationships between serum MK7 concentrations and mineral status of the skeleton in LP (30 mg)-treated rats **(G)** and in LP (100 mg)-treated rats **(H)**; and serum MK4 levels and BMD values of lumbar spine in rats obtained vehicle **(I)**. Legend to Figure 4: Data are shown as mean ± standard deviation (SD) - panels **(A–F)**, and Spearman’s correlation–panels **(G–I)**. *p < 0.05, **p < 0.01, ***p < 0.001; CON, controls; LP (30 mg), the group treated with LP at the dose of 30 mg/kg; LP (100 mg), the group treated with LP at the dose of 100 mg/kg; BMC, bone mineral 2content; BMD, bone mineral density; MK7, Menaquinone 7; MK4, Menaquinone 4.

We previously reported that LP administration restored femoral bone densitometric parameters in CKD rats, while VEH exerted a partial effect (Pawlak et al., 2018). In this study, we extended our analysis to the tibia and lumbar spine, the two skeletal sites representing bone with a greater content of cortical tissue (tibia) and trabecular tissue (lumbar spine) (Ott, 2018). As has been presented in Figure 4, CKD significantly reduced BMC and BMD, particularly in the trabecular-rich bone compartment (Figures 4B,C,E,F) compared to CON, while bone mineral area remained unchanged in cortical bone (Figure 4A) but was reduced in trabecular bone (Figure 4D). LP (100 mg) restored tibial BMC and BMD to control levels. Interestingly, the LP (30 mg) group exhibited even higher BMC and mineral area than both CON and LP (100 mg) groups, indicating a dose-dependent effect (Figures 4B,C). VEH treatment partially improved these parameters (Figures 4A–C). In the lumbar spine, CKD markedly impaired densitometric values. LP (30 mg) and VEH restored the BMC and mineral area to control levels. However, at the LP (100 mg) group, a decrease of these parameters was observed compared to both CON and LP (30 mg), though BMD remained unchanged (Figures 4D–F).

MK7 levels correlated positively and strongly with lumbar and tibial densitometric parameters in LP (30 mg) group (Figure 4G), whereas these correlations were weaker in the LP (100 mg) group (Figure 4H). In VEH, the MK4 level was related to BMD of the lumbar spine (Figure 4I), whereas there was no association between MK7 and densitometric parameters in both bone sites in this group.

### 3.5 The mutual relationships between the mineral status of the skeleton, *VKORC1* gene expression, and Gla-OC/Glu-OC ratios in the bone of CKD rats treated with LP533401

**FIGURE 5:**
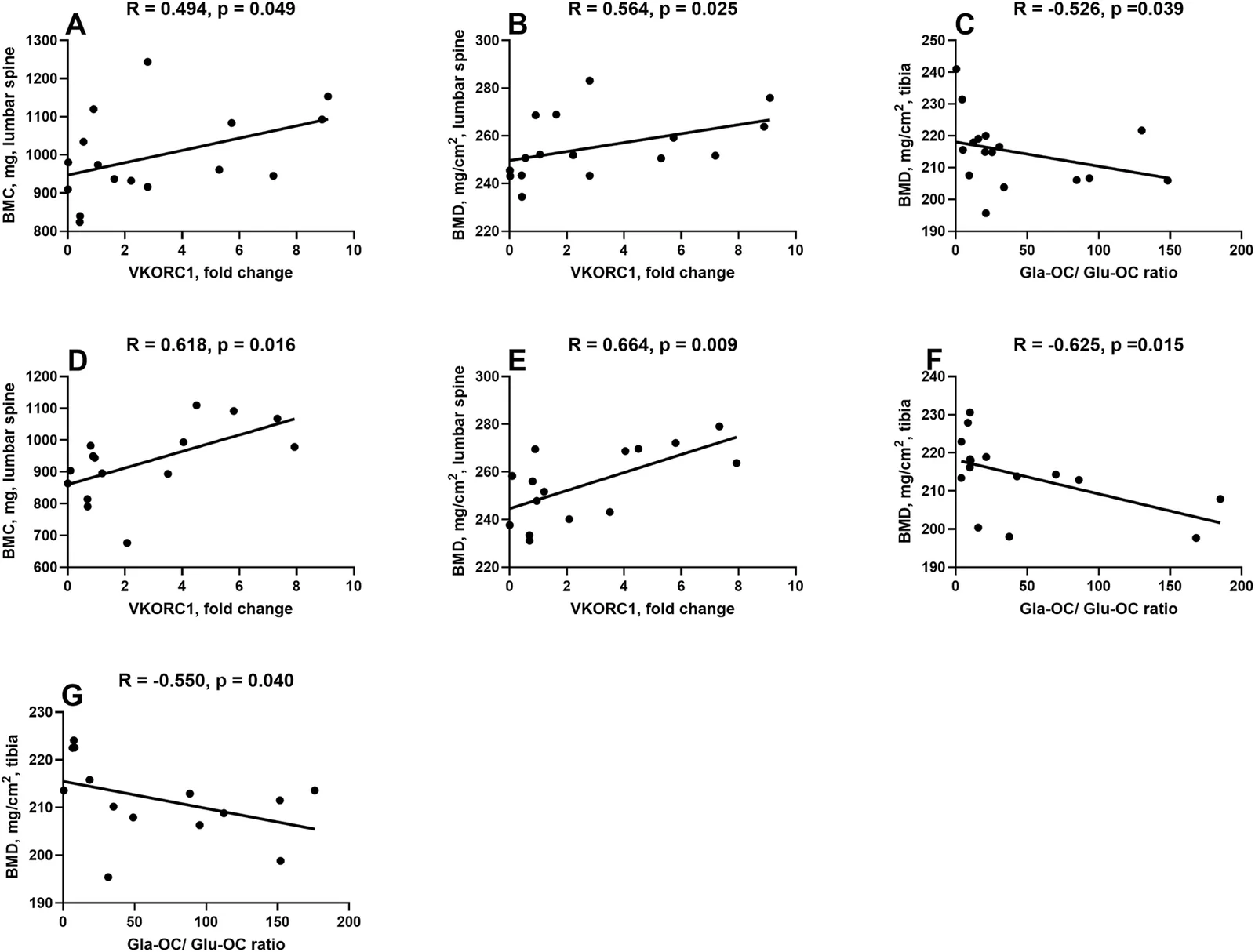
The positive associations between *VKORC1* gene expression and BMC, BMD values of lumbar spine in LP (30 mg) **(A,B)** and in LP (100 mg) group **(D,E)**, and the inverse relationship between trabecular Gla-OC/Glu-OC ratios and BMD values of tibia in rats with chronic kidney disease (CKD) treated with LP (30 mg) **(C)**, LP (100 mg) **(F)** or administered with vehicle **(G)**. Data are shown as Spearman’s correlation. BMC, bone mineral content; BMD, bone mineral density; *VKORC1,* vitamin K epoxide reductase complex subunit 1; Glu-OC, undercarboxylated osteocalcin; Gla-OC, carboxylated osteocalcin.

To explore whether systemic alterations associated with CKD and the investigated intervention are reflected across different skeletal compartments, correlation analyses were performed between molecular parameters assessed in femoral bone and DXA-derived outcomes obtained from the tibia and lumbar spine. Among the genes belonging to the VK cycle, only *VKORC1* expression positively correlated with lumbar spine BMC, BMD values in LP (30 mg) group (Figures 5A,B) as well as in LP (100 mg) group (Figures 5D,E), with a weak, non-significant trend in VEH (R = 0.436, p > 0.05). Given that these measurements were obtained from anatomically and functionally distinct skeletal sites, this analysis was designed as exploratory rather than to infer direct mechanistic relationships. The trabecular Gla-OC/Glu-OC ratios negatively correlated with tibial BMD in LP (30 mg) (Figure 5C), LP (100 mg) (Figure 5F) and VEH group (Figure 5G). Additionally, cortical Gla-OC/Glu-OC ratios tended to inversely associate with lumbar BMD in VEH-treated animals (R = −0.488, p = 0.076), indicating possible region-specific roles in bone mineralization.

## 4 Discussion

This study extends our previous work (Pawlak et al., 2018), in which we investigated the effects of LP533401 (LP), a gut-specific tryptophan hydroxylase 1 (TPH1) inhibitor, on CKD-induced skeletal abnormalities. The present study demonstrates for the first time that LP treatment modulates vitamin K (VK) metabolism, regulates the bone-specific VK cycle, affects functional biomarkers of VK status in bone and serum, and influences mineralization of distinct skeletal compartments in a rat model of CKD. Overall, our findings suggest that peripheral serotonin inhibition alters VK homeostasis and may contribute to the skeletal benefits previously observed with LP treatment.

Previous study has shown that CKD profoundly alters VK homeostasis by promoting the conversion of phylloquinone (VK1) into menaquinone-4 (MK4) and modifying VK recycling pathways in bone (Ziemińska et al., 2022). Our results are consistent with these observations. Untreated nephrectomized animals (VEH) exhibited the lowest circulating VK1 levels, markedly increased MK4 concentrations, and enhanced VK1-to-MK4 conversion efficiency. These changes were accompanied by increased expression of *UBIAD1, VKORC1*, and *GGCX*, key genes involved in VK metabolism and recycling. In particular, the elevated expression of *UBIAD1* in VEH rats supports enhanced extrahepatic conversion of VK1 to MK4 (Hirota et al., 2015) and may explain the predominance of MK4 over MK7 synthesis observed in this group.

LP treatment partially reversed these CKD-associated alterations in VK metabolism. Animals receiving LP showed reduced expression of *UBIAD1* and *VKORC1* compared with VEH animals. In addition, LP restored circulating VK1 levels and promoted MK7 formation, which is a long-chain menaquinone with superior extrahepatic bioavailability and activity (Sato et al., 2020). Consequently, MK7/VK1 and MK7/MK4 ratios increased, suggesting a shift toward a more favorable VK profile. Interestingly, *GGCX* expression normalized at the lower LP dose (30 mg) but increased again at the higher dose, indicating the existence of a dose-dependent regulatory response within the VK cycle. The mechanisms responsible for these effects remain speculative. Because LP inhibits peripheral serotonin synthesis, its actions may involve serotonin-dependent regulation of intestinal and hepatic metabolic pathways. Reduced serotonin signaling has been linked to changes in gut microbiota composition, intestinal absorption, and hepatic metabolism (Wen et al., 2024), all of which may influence VK homeostasis. Since MK7 is predominantly produced through microbial fermentation (Schurgers and Vermeer, 2000), the increase in MK7 observed after LP treatment may reflect enhanced microbial production or improved intestinal uptake. However, these mechanisms were not directly investigated in the present study and require further research.

The skeletal consequences of altered VK metabolism were assessed through analysis of osteocalcin (OC) carboxylation. Vitamin K serves as an essential cofactor for GGCX-mediated γ-carboxylation of OC, converting glutamate (Glu) residues into γ-carboxyglutamate (Gla) residues (Atkins et al., 2009; Roy et al., 2001). In VEH animals, both trabecular and cortical bone exhibited elevated Gla-OC levels, increased Gla-OC/Glu-OC ratios, and reduced Glu-OC concentrations, indicating enhanced VK-dependent carboxylation. Similar findings were previously observed in CKD animals (Ziemińska et al., 2022). The response to LP treatment differed between skeletal compartments. In trabecular bone, the lower LP dose increased the Gla-OC/Glu-OC ratio, whereas the higher dose restored this parameter to values close to those observed in control animals, suggesting partial normalization of γ-carboxylation efficiency. Moreover, the positive correlation between Gla-OC and Glu-OC indicates coordinated regulation of VK-dependent proteins in this metabolically active compartment. In contrast, cortical bone showed only modest changes following LP treatment. Glu-OC levels remained relatively stable, Gla-OC increased slightly, and the Gla-OC/Glu-OC ratio decreased without fully normalizing. These findings suggest that cortical bone is less responsive to LP-induced alterations in VK metabolism, likely reflecting its lower turnover rate and metabolic activity compared with trabecular bone (Ott, 2018). The absence of a significant correlation between Gla-OC and Glu-OC in cortical bone further supports compartment-specific regulation of VK-dependent processes.

To evaluate systemic VK status, we analyzed serum Glu-OC and undercarboxylated matrix Gla protein (ucMGP), both commonly regarded as indirect biomarkers of VK deficiency (Kremer et al., 2021; Silaghi et al., 2019; Fusaro et al., 2017; Neradova et al., 2022). Elevated serum Glu-OC levels were observed in all nephrectomized groups regardless of treatment. However, increased serum Glu-OC in CKD does not necessarily indicate VK deficiency alone. We previously demonstrated that accumulation of osteocalcin due to impaired renal clearance and secondary hyperparathyroidism may contribute to elevated circulating Glu-OC concentrations (Ziemińska et al., 2022). Indeed, parathyroid hormone can stimulate osteoblastogenesis and osteocalcin synthesis (Harsløf et al., 2015; Maser et al., 2018). In contrast, ucMGP levels remained stable or decreased following LP treatment, particularly at the higher dose. Furthermore, the inverse correlation between ucMGP concentrations and *VKORC1* expression suggests that enhanced local VK recycling may improve systemic VK-dependent carboxylation. This interpretation is consistent with previous reports demonstrating a critical role of *VKORC1* in regenerating active VK for *GGCX*-mediated protein carboxylation (Tie and Stafford, 2016; Ferron et al., 2015).

To determine the functional consequences of these metabolic changes, we evaluated bone mineralization in two anatomically distinct skeletal regions: the cortical bone-rich tibia and the trabecular bone-rich lumbar spine. Consistent with the known detrimental effects of CKD on the mineral status of the femur (Pawlak et al., 2018; Supplementary Table 1), nephrectomized animals exhibited reduced bone mineral content (BMC) and bone mineral density (BMD) at both sites. Bone mineral area (BMA) remained unchanged in the tibia but decreased in the lumbar spine, confirming impaired mineralization, particularly within trabecular bone. LP treatment exerted site- and dose-dependent skeletal effects. In the tibia, LP administered at 100 mg restored BMC and BMD to control values, indicating substantial protection of cortical bone. Interestingly, the lower dose produced even greater improvements in BMA and BMC, suggesting the existence of a therapeutic window. Vehicle treatment also improved tibial mineralization compared with untreated CKD animals, consistent with our previous observations in the femur (Pawlak et al., 2018; Supplementary Table 1), although its effects were less pronounced than those of LP. In the lumbar spine, both VEH and LP (30 mg) restored BMA and BMC, whereas LP100 unexpectedly reduced these parameters despite maintaining normal BMD values.

The relationship between VK metabolism and skeletal outcomes was further supported by correlation analyses. MK7 concentrations showed positive associations with densitometric parameters, particularly in LP (30 mg)-treated animals, and weaker but similar relationships in LP (100 mg)-treated rats. These findings support a beneficial role of MK7 in bone mineralization. In contrast, MK4 correlated only with lumbar BMD in VEH animals, suggesting a more limited contribution to skeletal health. The markedly lower MK4 levels observed in LP (100 mg) animals may partly explain the unexpected reduction in lumbar BMA in this group. Notably, the absence of significant associations between MK7 and bone parameters in VEH animals suggests that the skeletal benefits of MK7 may become more apparent in the context of serotonin inhibition.

Among the VK-related genes examined, only *VKORC1* expression was positively associated with lumbar BMC and BMD in LP-treated animals, supporting its role in VK-dependent protein activation and mineral accrual. However, the observation that higher Gla-OC/Glu-OC ratios were associated with lower tibial BMD indicates that VK-dependent carboxylation alone cannot fully explain the mineralization phenotype. Similarly, VEH animals exhibited high *VKORC1* expression and elevated Gla-OC/Glu-OC ratios despite reduced BMD. Since these animals also displayed elevated PTH concentrations (Pawlak et al., 2018; Supplementary Table 1), increased osteocalcin carboxylation may reflect enhanced bone turnover rather than improved skeletal quality (Weng et al., 2019; Ziemińska et al., 2022). This interpretation is consistent with studies proposing that osteocalcin, particularly its carboxylated form, may function as a negative regulator of bone mass under specific physiological conditions (Ducy et al., 1996; Lambert et al., 2016; Simon et al., 2018).

Although the present findings support an association between peripheral serotonin inhibition, vitamin K metabolism, and skeletal mineralization in CKD, they do not establish a direct causal mechanism linking these processes. Rather, the proposed peripheral serotonin–vitamin K axis should be regarded as a mechanistic hypothesis supported by the current data, which requires validation in dedicated mechanistic studies.

A major strength of this study is the comprehensive assessment of VK metabolism, expression of VK cycle enzymes, and VK-dependent functional biomarkers not only in serum but also directly in the relevant bone compartments. The main limitations are its cross-sectional design, which is exploratory in nature and does not permit conclusions regarding causal relationships. The observed correlations between molecular parameters measured in femoral bone and DXA-derived outcomes from the tibia and lumbar spine should be interpreted with caution. These skeletal sites differ in terms of microarchitecture, remodeling dynamics, and mechanical loading, which may influence their response to CKD and therapeutic intervention. Future studies employing site-matched analyses will be necessary to confirm and further elucidate these relationships. Another limitation is the incomplete inclusion of the untreated CKD group across several analyses. Data from untreated CKD animals have been partially reported in our previous publication (Ziemińska et al., 2022), what limited our ability to fully re-present these data in the current study. Consequently, comprehensive comparisons between treated and untreated CKD animals were not possible for all analyses, and this should be considered when interpreting the observed treatment effects. In addition, other factors that may influence the analyzed parameters were not investigated in the present study.

## 5 Conclusion

Building upon previous evidence that LP533401 reduced serotonin turnover and improved bone mineral status, microarchitecture, and strength in rats with CKD (Pawlak et al., 2018), the present study extends these findings by demonstrating that LP533401 is associated with changes in vitamin K metabolism, regulation of the vitamin K cycle, bone and systemic biomarkers of vitamin K status, and vitamin K-dependent bone mineralization. Collectively, these findings support the hypothesis that modulation of the peripheral serotonin–vitamin K axis may contribute to improvements in skeletal mineral status in CKD, although the underlying mechanisms remain to be fully elucidated. The observed dose-dependent effects of LP533401, whereby the 30 mg dose produced more pronounced improvements in selected bone mineral parameters than the 100 mg dose, further indicate that optimization of therapeutic strategies targeting this pathway warrants future investigation.

From a translational perspective, our findings suggest that modulation of vitamin K metabolism, particularly with respect to MK7, warrants further investigation as a potential strategy to support skeletal health in CKD-MBD. However, these observations are based on an experimental animal model and require confirmation in dedicated mechanistic and clinical studies before any therapeutic implications can be drawn.

In summary, our findings provide further evidence supporting an interaction between peripheral serotonin, vitamin K metabolism, and skeletal mineralization in CKD. These results contribute to a better understanding of the complex interplay between these pathways and provide a rationale for future studies aimed at elucidating the underlying mechanisms and evaluating their potential clinical relevance.

## Data Availability

The original contributions presented in the study are publicly available. This data can be found here: https://doi.org/10.6084/m9.figshare.33340461.
