# Supplementary material for: LP533401 treatment alters vitamin K metabolism, bone Gla-OC/Glu-OC ratios, and improves mineral status of the skeleton in rats with chronic kidney disease
Source: Front Pharmacol. 2026 Sep 4;17:1793663. doi: 10.3389/fphar.2026.1793663 (PMC13585592; doi:10.3389/fphar.2026.1793663)
Supplement: Supplementary file 1 [file Table1.docx]

Supplementary Table 1. Schematical presentation of changes in basic parameters across all experimental groups.

| Parameter | CKD,  n=16 | VEH, n=14 | LP (30 mg),  n=16 | LP (100 mg), n=15 |
| --- | --- | --- | --- | --- |
| Body weight, g | ↓↓↓ | NS, ** | ↓, * | ↓, * |
| Body length, cm | NS | NS | NS | NS |
| Creatinine, mg/dl | ↑↑↑ | ↑↑↑ | ↑↑↑ | ↑↑↑ |
| Creatinine clearance, ml/min | ↓↓ | ↓ | ↓↓ | ↓↓ |
| Urea, mg/dl | ↑↑↑ | ↑↑↑ | ↑↑↑ | ↑↑↑ |
| Pi, mg/dl | ↑↑ | ↑ | ↑ | NS |
| PTH, pg/ml | ↑↑ | ↑↑↑, ** | ↑↑↑, * | ↑↑↑, ^ |
| Femoral BMA, cm^2^ | ↓ | * | ** | # |
| Femoral BMC, mg | ↓↓ | ** | *** | * |
| Femoral BMA, mg/cm^2^ | ↓↓ | * | ** | ** |

For detail please see Pawlak et. al., 2018

↓/↑ indicates the direction of change relative to the control group; ↓/↑ p < 0.05, ↓↓/↑↑ p < 0.01, ↓↓↓/↑↑↑ p < 0.001; NS – non statistically significant

*p<0.05, **p<0.01, ***p<0.001 indicates the reduction in CKD versus others; ^p<0.05 indicates the reduction in LP (100 mg) versus VEH; # p<0.05 indicates the reduction in LP (100 mg) versus LP (30 mg)

Abrreviations: CKD – chronic kidney disease; VEH – vehicle, LP (30 mg) – 30 mg/kg LP533401, LP (100 mg) – 100 mg/kg LP533401; PTH – parathyroid hormone; BMA – bone mineral area; BMC – bone mineral content; BMD – bone mineral density
